# ASSOCIATION OF ATTITUDES WITH ECONOMIC WELL-BEING AND HEALTH: A FOUNDATION FOR PUBLIC POLICY

**DOI:** 10.1093/geroni/igz038.456

**Published:** 2019-11-08

**Authors:** Sarah B Laditka, James N Laditka, Laura Gunn

**Affiliations:** University of North Carolina at Charlotte, Charlotte, North Carolina, United States

## Abstract

The 1960s War on Poverty was based on expectations that certain attitudes could improve health and economic well-being: aspiration and ambition, propensity to plan, personal efficacy, avoidance of unnecessary risk, connectedness to information and help, and trust. If true, promoting those attitudes might improve lives. The nationally representative Panel Study of Income Dynamics (PSID) developed scales to repeatedly measure associations of those attitudes with income and well-being. After five annual measurements, researchers found few associations. Acknowledging more data might be needed, researchers concluded that changing attitudes was unlikely to help. We studied those same associations using five decades of PSID measures on income and work disability, physical or “nervous” health problems limiting work (1968-present; n=5,027; 170,583 person-years; mean baseline age 34.2), with multinomial logistic Markov models and dynamic microsimulation, modelling three levels of each outcome plus death. We also examined persistence of the attitudes (measurement reliability). Results suggested the attitudes were persistent (intraclass correlations > 0.87). Controlling for age, sex, race, education, and baseline income, attitudes were strongly associated with the outcomes. For example, with above-median baseline income, 19.4% in the top baseline self-efficacy quintile had incomes below 150% of poverty at age 70, compared with 27.0% in quintile 1 (p<0.001). Similarly, 5.5% in the highest quintile reported severe work disability (could not work, or limited “a lot”) at age 70 compared to 23.7% in the lowest (p<0.001). Other attitudes showed similar significant patterns. Attitudes in early- to mid-adulthood may contribute importantly to economic well-being and health throughout later life.

